# Individualized Health Care for Older Diabetes Patients from the Perspective of Health Professionals and Service Consumers

**DOI:** 10.3390/jpm11070608

**Published:** 2021-06-27

**Authors:** Birute Bartkeviciute, Vita Lesauskaite, Olga Riklikiene

**Affiliations:** Faculty of Nursing, Lithuanian University of Health Sciences, LT 44307 Kaunas, Lithuania; vita.lesauskaite@lsmuni.lt (V.L.); olga.riklikiene@lsmuni.lt (O.R.)

**Keywords:** individual care, nurses, older diabetes patients, physicians, support

## Abstract

Background: Individualized nursing care as a form of person-centered care delivery is a well-known approach in the health care context and is accepted as best practice by organizations and professionals, yet its implementation in everyday practice creates serious challenges. The aim was to assess and compare the perceptions of health professionals and older diabetes patients on their individual care in regard to the patient’s clinical situation, personal life situation, and decisional control. Methods: The quantitative study with a cross-sectional survey design was conducted from March 2019 until January 2021. The Individualized Care Scale was applied for the data collection. Health professionals (nurses and physicians, *n* = 70) and older diabetes patients (*n* = 145) participated in the study. The average duration of diabetes was 15.8 years (SD = 10.0) and type 2 diabetes was the most common (89.0%). The current glucose-lowering therapy for 51.0% of the patients was oral medications, 37.9% used injected insulin, and 11.1% were treated by combined therapy. Results: The highest-rated aspects of individualized care on both dimensions of the scale from the health professionals’ perspective related to the clinical situation, and the scores for provision were significantly higher than those for support. The highest means of patients’ ratings on the support dimension related to the clinical situation and the decisions over care sub-scale; for the care provision dimension, the highest individuality in care was assigned to the decisions over care sub-scale. The lowest ratings of individualized care, both in the health professionals’ and patients’ samples, related to the personal life situation sub-scale. Conclusions: Health professionals are more positive in regard to individualized care support and provisions for older diabetes patients than the patients themselves. Patient characteristics, such as the type of glucose-lowering therapy, education, and nutritional status, make a difference in patients’ understanding and experience of individuality in care.

## 1. Introduction

Patient-centered care is a well-known and widely used approach in the health care context, as well as in education, management, and scientific investigations. The WHO global strategy on people-centered and integrated health services (2015) highlighted the importance of placing people and communities at the center of health services, which makes health services more comprehensive and responsive, more integrated, accessible, coordinated, safe, more focused on health needs and preferences, and more humane and holistic. Patient-centered care is understood as health services throughout the course of life that respond to the consumers’ values and preferences, and are ‘organized around the health needs and expectations of people rather than diseases’ [1], p. 7.

A particular advantage of patient centralization in care, as mentioned by the WHO strategy, is an improved ability to respond to health care crises. Now, health care systems and societies, still guided by the global pandemic, have never been more clearly aware of the importance of patient-centered care for individuals’ positive health outcomes and high-quality and continuity of care. As it was stated in the WHO document, a well-organized health system that is ‘able to adapt to the needs of the people it serves is not only better positioned to respond to emerging threats, but is also more resilient to tackling the myriad of chronic diseases which plague our populations’ [1], p. 5.

Several studies and reviews addressed care individuality, a form of person-centered care delivery, for chronic disease patients. The evidence was created on preferences for decision-making among chronic neurodegenerative disease (Parkinson’s) patients [2] and the benefits of nurse-led patient education for adults with heart failure [3,4]. Nurse-led case management was tested on adults with cancer [5] and other chronic illnesses [6]. The effectiveness of community-based case management among adults who abuse substances [7] and nurse-led primary health care coordination were investigated for patients with complex needs [8]. The results of these studies indicated multidisciplinary teamwork, the delivery of personalized, integrated, and coordinated care, patient education, and self-management skill improvement as suitable strategies in solving complex patients’ health and social care needs in the primary health care of chronic illnesses, including diabetes mellitus.

The number of new diabetes cases has been increasing globally, with a projected increase to 26.6 million cases of incidence, 570.9 million cases of prevalence, and 1.59 million deaths in 2025 without effective interventions [9]. There were 109,162 diabetes mellitus cases (0.8% were children) in Lithuania in 2018, that is, 1 in every 28 men and 1 in every 23 women. Complications of the illness were prevalent in 80.5% of patients with type 1, and in 46.1% of type 2 diabetes patients; 528 patients die annually from this disease or its complications. In 2019, the number of patients with diabetes increased to 110,136 (396.3/1000 pop.); 6421 (2.3/1000 pop.) had type 1 diabetes and 104,659 (37.46/1000) were ill with type 2 diabetes [10].

Health care service for patients with diabetes, besides the physical, psychological, and social consequences, brings remarkable financial expenses, creating an economical burden for national health care systems. In 2011, estimates of global health care expenditures due to diabetes were USD 376 billion, and for the whole European region, it was USD 105 billion (10% of total health care expenditures; USD 2046 per person), with expectations for it to increase to USD 490 billion by 2030 [11]. A study conducted by Domeikiene et al. (2014) calculated the direct health care costs needed for patients with type 2 diabetes mellitus (non-complicated and complicated cases) in Lithuania. The results revealed that costs, adjusted for the average annual cost per person with diabetes, increased gradually with the number of complications from USD 814.73 (95% CI, 697.22–932.24) in patients without complications to USD 1926.64 (95% CI, 1275.66–2577.61) in patients with three or more complications [12].

There is evidence that patient-centered care positively relates to reduced pain and discomfort, faster physical and emotional recovery, improved outcomes and quality of life [5,13], enhanced respect for persons and autonomy [2], increased adherence to the care plan [14], reduced hospital readmissions [3,13], and decreased health care utilization [15]. Similar patient health and care issues are intrinsic to older diabetes patients.

Various care strategies were suggested to improve the care and quality of life for patients with diabetes. In nursing, traditional nursing theoretical frameworks with patient education strategies were utilized for improving the care and self-care behaviors of people with diabetes [16,17,18,19]. To improve care for diabetes patients, an order of the Health Ministry on the requirements for the provision of nursing services for patients with diabetes mellitus was issued in Lithuania. The order states the right for diabetes nurses to provide primary and continuous consultations, and perform foot and leg ulcer care for patients independently or with a team [20]. Patient education becomes an important part of diabetes nurse service, as it encourages behavior changes in people with diabetes, which, in turn, may improve their chances for diabetes control. The clinical trial on continual diabetes education confirmed that values and achievement of the type 2 diabetes mellitus control improved after face-to-face individualized education sessions for patients [21]. During individual and group education, general information is provided, trying to correspond to the basic needs of the patients, enabling them to be active participants and creating a possibility to learn from the experiences of others. However, diabetes patients, particularly those of older age, are not always prepared to take an active role when discussing their care with health care professionals as they are lacking skills [22] or are not familiar with what their exact role in that discussion should be.

Even if the approach of patient-centered care is familiar to health care organizations and health professionals, the implementation of such care commonly creates challenges. The obstacles are related to the differences in the comprehension of the phenomenon, a necessary physical change of health facilities, a serious cultural shift of the paradigm in traditional care practices (e.g., a paternalistic approach vs a consumer-oriented approach to care), organizational initiatives, leadership capacities, and health care professionals’ knowledge and skills [23,24]. To tackle the situation, it is important to analyze the providers’ perspectives on the support and provision for individual care for patients. In addition, the consumers’ feedback of their experiences in patient-centered care may be of great benefit for the improvement of the work at health care organizations [25].

To our knowledge, there is a lack of studies that explore older diabetes patients’ perspectives on individualized care and compare the experiences of patients with those of health professionals (nurses and physicians). For the Republic of Lithuania and similar post-soviet states that began to transform the national health care system in the early 1990s by adopting the Western countries’ principles of health service provision and modern nursing developments, the individualized approach to the care of older patients with chronic disease is a new practical reality where more evidence is needed. The results of this study highlight the need for more active involvement of older diabetes patients in care planning and provisions by communicating their preferences and making individual decisions. We also expect that increasing the data on Lithuanian nurses’ perspectives on individualized care for diabetes patients will facilitate change by fostering the development of the independent role and expanding the competence of diabetes nurses in other countries where such specialty has not been introduced yet.

The aim of this study was to assess and compare the perceptions of health professionals and older diabetes patients on their individual care in regard to their patient clinical situation, personal life situation, and decisional control.

## 2. Materials and Methods

### 2.1. Design and Setting

A quantitative study with a cross-sectional survey design was conducted. The study was performed from March 2019 until January 2021.

The study was conducted at the primary health care institutions (*n* = 10) of the Kaunas region (i.e., the second largest city of Lithuania), where diabetes care for patients is provided by primary care physicians, physician endocrinologists, diabetes nurses, and general practice nurses. All institutions are funded by the national compulsory health insurance fund. Before the pandemic, questionnaires were distributed by the investigator when she met nurse managers at the study field, introduced the study, and asked for help in distributing and collecting the questionnaires to health professionals and patients. Patients, in most cases, picked up the questionnaire with a stamped envelope during their regular check-up, completed it at home, and returned it during the next visit or by mail. At the time of the pandemic and quarantine, health professionals were addressed through their work emails and were sent an e-questionnaire. The patients were not surveyed by e-mail.

### 2.2. Instruments

For this study, 2 versions of the Individualized Care Scale (ICS) [26,27,28,29] were applied for the data collection. The Nurse Version (ICS–Nurse) was filled in by the nurses and physicians who take care of older diabetes patients, and the Patient Version (ICS–Patient) of the instrument was delivered to older patients with either type 1 or type 2 diabetes mellitus.

The Individualized Care Scale–Nurse Version (ICS–Nurse) is a bipartite questionnaire designed to explore nurses’ views about individualized care in two dimensions (A-ICS–Nurse and B-ICS–Nurse) [29]. The current version of the scale includes 17 (A) and 17 (B) items, for a total of 34. The A-ICS–Nurse is a 17-item 5-point Likert-type scale (1 = strongly disagree, 2 = disagree to some extent, 3 = neither agree nor disagree, 4 = agree to some extent, 5 = strongly agree) designed to explore nurses’ views on how they support patient individuality through nursing activities in general. For example, the nurse is asked to rate the items ‘I talk with patients about their fears and anxieties’ or ‘I help patients take part in decisions concerning their care’. The B-ICS–Nurse is also a 17-item 5-point Likert-type scale exploring the extent to which nurses perceive that the care they provide is individualized (e.g., ‘I took into account their needs that require care and attention’) [30,31]. Both scales consist of 3 sub-scales: (1) clinical situation (Clin A and B), (2) personal life situation (Pers A and B), and (3) decisional control over care (Dec A and B).

The Individualized Care Scale–Patient Version (ICS–Patient) is a 34-item self-reporting measure that was developed for the purposes of exploring patients’ views on how patient individuality was supported through specific nursing activities (A-ICS–Patient) and the extent to which patients perceived their care as individualized (B-ICS–Patient) [27].

Both dimensions consist of 3 sub-scales eliciting information on the following: (1) patient characteristics in the clinical situation caused by hospitalization (or, for this study, a visit to an outpatient clinic) (Clin A and B), (2) the patient’s personal life situation (Pers A and B), and (3) decisional control over care (Dec A and B). Both dimensions, that is, A and B-ICS–Patient, share the same structure in terms of their content. The questions are differently worded in that some A-ICS–Patient items ask the patients how nurses’ activities have facilitated their individual existence in care, whereas some B-ICS–Patient items present the question as to how individual or individualized the care for the patients has been. The following response categories were used: 1 = fully disagree, 2 = disagree to some extent, 3 = neither disagree nor agree, 4 = agree to some extent, and 5 = fully agree. Higher scores indicated higher individuality in care from the patient perspective. 

The ICS–Nurse and ICS–Patient versions have been previously translated and validated in various European countries with nurses of different specialties (e.g., general surgery, orthopedic surgery, maternity ward, geriatric, rehabilitation) and patients (e.g., patients from surgical, internal medicine, oncological, and gynecological wards) [28,29,30,31,32,33]. For this study, the Individualized Care Scale (ICS) was forward-translated into the Lithuanian language and back-translated into English following the methodological considerations for double translation and reconciliation [34].

To assess the psychometric properties, Cronbach’s alpha was calculated for internal consistency for both scales (ICS-A and ICS-B) and 6 sub-scales of both instruments, for nurses and patients. In this study, 1 item from the sub-scales A and B (No. 17) that related to the patient’s preference of washing time in a day was excluded from the Lithuanian version of the Individualized Care Scale (ICS) in the Nurse and Patient Versions, as it was not relevant for the primary health care service profile.

The 2 scales and 6 sub-scales of the Individualized Care Scale–Nurse Version (ICS–Nurse) had Cronbach’s alpha (α) values ranging from 0.79 to 0.92; the Cronbach’s alpha (α) for the Individualized Care Scale–Patient Version (ICS–Patient) ranged from 0.77 to 0.96, indicating appropriate (from satisfactory to excellent) internal consistency of the instrument for both samples (Table 1). When compared with the Dutch validation study, we observed that the internal consistency of the Lithuanian version of the ICS was weaker [33].

The Individualized Care Scale–Nurse Version (ICS–Nurse) was used for health care professionals that provide care for older diabetes patients. Even if the ICS is primarily aimed at nurses’ support and provision of individualized care, we used this scale for physicians as well. By not separating physician and nurse contributions to individualized care, we argue that nurses and physicians work as a team while providing care for older patients with diabetes. Thus, the team is responsible for finding the means to assure that their care plan implements each patient’s preferences and enables him/her to actively participate in their care by making decisions about their own care. Moreover, the scale items appear to be generic (not specific to only nursing activities) and ask patients to respond about general features of an individual approach to care. It is also expected that the study results will help to detect the weakest points of patient-centered care implementation for the specific population of our study, that is, older diabetes patients, and will serve as a basis for further joint educational initiatives for both physicians and nurses.

The following background data about the nurses’ and physicians’ characteristics were requested from the participants: age, gender, education, occupation/job title (nurse/physician), and years of professional experience. In addition, the patients were asked to provide their age, gender, education level, height and weight, type of diabetes, most recent HbA1c, and duration of illness.

### 2.3. Participants

Nurses and physicians (*n* = 126, response rate—96.9%), as well as older patients with diabetes (*n* = 145, response rate—72.5%), participated in the anonymous survey. The enrollment criteria for the study for nurses and physicians was the provision of care for older diabetes patients for more than one year. Every nurse and physician from the institutions involved in the study that suited the inclusion criteria were invited to participate in the study.

Among the health care professionals (N = 70), the majority were nurses (81.4%, 57) and the others were physicians (28.6%, 13). Regarding gender, all the respondents were female. The mean age of the respondents (nurses and physicians) was 48.57 ± 9.33 years (range of 24–65, median of 49.5 y.). More than half of the health care professionals (65.7%, 46) had completed higher education (university or college), and the others (34.3%, 24) had received a vocational education (nursing school with a diploma). The average duration of professional experience was 24.37 ± 10.96 years (range of 1–43); 34.3% worked in their professional practice for fewer than 20 years, and the others (65.7%, 46) for 20 years or more.

Patients were consecutively enrolled based on the following criteria: age of 65+, with a confirmed diagnosis of type 1 or type 2 diabetes mellitus, with more than 1 year of the disease’s duration, having Lithuanian language reading and writing skills, and being able to comprehend the questions. Each older diabetes patient that visited a primary health care center or outpatient unit of the hospital during the study period (23 months in total) was invited to participate in the study. Those who declined to answer the questionnaire explained being busy at the time, not being interested in the study, or having vision or Lithuanian language problems. The characteristics of the patient sample are presented in Table 2.

### 2.4. Ethical Considerations

Permission to conduct the study was obtained from the Kaunas Regional Biomedical Research Ethics Committee on 13 March 2019, No. BE-2-29.

### 2.5. Statistical Data Analysis

The data were analyzed using the Statistical Package for Social Sciences (IBM SPSS Statistics) version 25.0. To assess the psychometric properties of the scale, Cronbach’s alpha was calculated for the internal consistency of the individual items and the sub-scales; the internal consistency of α > 0.6 was considered to be acceptable [35].

The results were presented in percentages for the qualitative variables and the means with standard deviation (SD) were calculated for the quantitative variables. For the ICS, the higher the mean scores, the better patient individuality was supported (ICS-A–Nurse) and the higher the perceptions were of the maintenance of individuality in care (ICS-B–Nurse).

The nonparametric Kolmogorov–Smirnov normality test was used to test the normal distribution of the data. As normality was absent, the nonparametric Mann–Whitney U test was used to compare the distributions of the quantitative variables for the independent groups. The Wilcoxon signed-ranks test was applied to compare the results from the 2 ICS dimensions. The significance of the differences was defined by a *p*-value of <0.05.

## 3. Results

At the sub-scale level, health professionals scored the perception of individual care provided to the patient (B-ICS–Clinical) (mean 4.17) and views on support to the patient (A-ICS–Clinical) (mean 4.13) at clinical situations as the highest. The lowest professionals’ and patients’ scores in both domains (support and provision) of individualized care were associated with the patient’s personal life situation (Table 3). Accordingly, the highest means of patients’ ratings of individualized care in the support dimension related to the clinical situation (mean of 3.55) and decisions related to care (mean of 5.54) sub-scales. For the care provision dimension, the highest individuality in care from the patients’ perspective was related to the decisions over care domain (mean of 3.65). Moreover, the ratings of support for individualized care and such care provision were significantly higher for the health professionals than for the patients in each sub-scale of the ICS (Table 3).

At the ICS item level, significant differences between the health professionals’ and patients’ perspectives towards the support of individuality in care were determined for the majority of the items (Part A); the scores of the health professionals were higher than those of the patients (Table 4). In contrast, no differences were revealed in the answers about previous experiences of hospitalization and patients’ participation in decision making.

Regarding the support scorings, the health professionals’ and patients’ perspectives on the provision of individual care for older patients also varied significantly; the professionals were more positive in their answers than the patients were (Table 5). The nurses and physicians thought similarly to the patients only in the cases of the patients’ chance to take responsibility as far as possible and their knowledge preferences (what they want to know about illness/health condition).

A comparative analysis of ratings among health professionals revealed statistically significant differences between the two dimensions of the ICS, that is, the professionals’ views on support to the patient through care activities and the professionals’ perception of individual care provided to the patients. The differences were related to the personal life situation and the decisional control over care sub-scales; the health professionals’ ratings of provision were significantly higher than those for support (*p* = 0.046 and *p* = 0.037, respectively, based on the Wilcoxon test).

Within the health professionals’ sample, there were no differences in the scores (neither at the dimension nor at the sub-scale level) of individualized care between physicians and nurses. The sociodemographic characteristics in the total sample of health professionals did not make any differences for individualized care scores as well.

As all the physicians (N = 13) had a higher university education, we separately analyzed the nurses’ (N = 57) ratings on the ICS sub-scales in relation to their education. Nurses who take care of older diabetes patients and who have had vocational education scored support for individual care (Part A) with significantly higher scores than nurses with a college education (the means were 4.11 and 3.68, *p* = 0.039, respectively,). In addition, it was observed that the tendency for nurses with a university education rated provision (Part B) higher than nurses with a college education; the means were 4.16 and 3.66, *p* = 0.052, respectively.

Several factors made a difference in the perception of individual care in older patients with diabetes. The highest differences in diabetes patients’ assessments of individualized care dimensions and sub-scales were observed in relation to the current type of glucose-lowering therapy. Patients with combined therapy rated each sub-scale of the provision dimension and particular sub-scales of the support dimension of individual care significantly higher than those with oral or injectable medications (Table 6).

In addition, patients with lower than a degree education level rated the support dimension (Part A) higher than those with a college or university education; the means were 3.60 and 3.12, *p* = 0.032, respectively.

Body mass index (BMI) was also a variable that resulted in variations in the patients’ perceptions of support for individual care. Older diabetes patients with a BMI of <30 rated the support dimension significantly lower than patients with a BMI of ≥30; the means for Part A of the ICS were 3.34 and 3.68 respectively (*p* = 0.032).

Moreover, a tendency was observed in relation to the patients’ ages and their opinions on individual care; patients of 71 years and over rated the support for their personal life situation (A-ICS–Personal) higher than those who were 70 years old or below (*p* = 0.054).

## 4. Discussion

A patient-centered approach to care sees individuals as active recipients of health care provision. Patients are expected to collaborate with medical professionals, discuss their clinical and life situations, and make decisions related to their care plan. Health professionals (nurses and physicians) taking care of older diabetes patients in the Lithuanian primary health care organization had an opinion that they support patient individuality, in general, and provide care that takes into account the particular patient’s situation. The highest scores from the professionals’ perspective were for the support and provision of individual care in regard to the clinical situation of the patient. These results generally correspond to the comparative cross-cultural study on individualized nursing care in seven countries [31]. Very similar nurses’ positive perceptions of individualized care, especially for the clinical situation and patients’ decisional control over care, were also found by Finnish researchers [36].

Notably, patients in our study rated the support for individual care higher than patients from internal medicine and surgical units of teaching hospitals in Turkey, although the results of both studies correspond in regard to the patients’ ratings of the personal life situation aspect of the support dimension, where the mean was the lowest [32]. Similar to a study on orthopedic and trauma patients [37], the personal life preferences of the patient were also at least discussed during care provision for the Lithuanian older diabetes patients from both patients’ and health professionals’ points of view.

In all cases, the ratings of support for individualized care and such care provision through care activities were significantly higher for the health professionals than for the patients. An international study among five European countries revealed similar differences in patients’ and nurses’ assessments of individualized nursing, where nurses, compared with patients, assessed that they supported patient individuality more often [30]. In Lithuania, the data about chronically ill patients’ preferences and readiness for active participation in care and in decision-making, particularly, is scarce and creates difficulties for a valid interpretation of the current results. The arguments for a lower rating of individual care support and provision in the patient sample might be twofold: either they wish but lack actual possibilities to participate, discuss, and make decisions, or their understanding and knowledge about such possibilities is incomplete and they are not even motivated to be an active participant in their care. Such an interpretation is consistent with the findings of du Pon et al. (2019), in which patients with diabetes were found to have limited necessary skills to be adequately prepared for a consultation and achieve an active role [22]. In prior research on the preferences and participation in decision-making among patients with Parkinson’s disease, the authors suggest that in some contexts or situations, patients prefer less autonomy in medical decision-making and choose shared decisions, or even find it acceptable to be excluded from decision-making as their illness worsens. Some patients preferred to make the final decision, some wanted the decision-making process to be evenly shared, while others preferred to delegate final decisions to the doctor [2]. Further research applying a rigorous quantitative and qualitative design to larger populations of diabetes patients is recommended. Future work should also address how different groups of patients understand and prefer individuality in care, and how this care is linked with other relevant factors.

The ratings of individual care for the ICS dimensions and sub-scales did not vary among health professionals in regard to their qualification, that is, being a nurse or physician. Although, education level made a difference in how nurses comprehend and practice their individual approach in the care of older diabetes patients, as the nurses with a vocational education were more positive and rated higher individual care proposed and provided for the older diabetes patients than the college degree nurses. These results are in contrast with those of Suhonen et al., (2009) who found that higher scores supporting the delivery of individualized care could have been expected from nurses with higher education [38]. Such results may be explained by the differences that were introduced in the nursing curriculum in the last decade, when nursing education was elevated from a diploma to a college or university-based system. As a condition for membership in the European Union (EU), nursing programs were harmonized with the EU requirements, which caused remarkable transitions of nursing education from a strongly biomedical, technical approach toward a more sensitive, patient-centered, holistic approach to care [39]. We propose that higher educated nurses are well equipped with the knowledge of modern nursing and have a clearer vision of what care should be in relation to safeguarding the patient’s individuality. This is the reason they are more critical in assessing the current situation, which is lacking a full integration of the patient-centered approach in older diabetes patient care practice.

Our results revealed that patients with combined glucose-lowering therapy rated some aspects of support and all the aspects of the provision of individuality in their care higher than those patients who were treated by oral or injectable medications. We propose that a complex type of treatment requires the physicians and nurses to give more attention to an individual patient′s case through collecting health information, assessing health status, instructing, and educating. In addition, the integration and monitoring of patient-reported outcomes facilitates holistic interdisciplinary care and takes into account patient-relevant endpoints [40]. Such a relatively prolonged communication and going into the situation creates for the patient an impression of greater consideration for his or her particular case. A similar interpretation might be true in regard to the body mass index, as diabetes patients with a BMI of ≥30 had more positive opinions about individual care support than those patients with a lower BMI.

Patients’ perceptions of individualized nursing care are related to their education level. In our study, older diabetes patients with a lower than degree education level rated the support dimension (Part A) higher than those with a college or university education. Other researchers similarly reported that a lower educational level is associated with a perception of more individualized care in patients [36,41].

The findings of this study provide the initial evidence of the perception and provision of the individualized care approach to the care of older diabetes patients. For improvements in clinical practice and fostering the actual implementation of a patient-centered approach of care, complex means would be the most effective for unifying professional, organizational, and policy development. The research showed that older patients’ perceptions of individuality in care were associated with the care environment, especially a patient-centered care climate [42]. In order to be responsible in personal care decisions and adhere to health recommendations (e.g., medications, nutrition, and physical activity), patients require adequate ‘education and support they need to make decisions and participate in their own care’ [1], p. 7. It might be assumed that continuous patient–provider communication, regular contact, and careful consideration of individual values, knowledge, habits, and behaviors motivate and empower older and chronically ill patients to change. Further continuing education of physicians and nurses, joining them in one class and with the same teaching content, would help to expand their unified understanding of patient-centered care components, principles, and practical implementation in caring for older diabetes patients.

This study was one of the first attempts to study individual patient care in our country. For further research, physicians’ and nurses’ work environment characteristics that can also affect the provision of individualized have to be considered [43]. There are more factors of the professional practice environment found that correlate with the nurses’ perceptions about the support of individuality and their views on the individual care provided [44]. This means that for a real shift toward an individual approach to care, health care institutions need to expand their philosophy of service to support the new roles of care providers and consumers at an organizational level.

This study has several limitations. Firstly, the Individualized Care Scale–Nurse Version (ICS–Nurse) was initially developed and validated to assess nurses’ points of view about individual care support and the provision of such care through nursing activities, particularly. The evidence for the validation of this scale among other health care professionals is lacking and should be separately addressed in further studies. Secondly, the construct of the scale (a bipartite structure with a rather similar wording of items for both dimensions) may influence the clear comprehension and accuracy of the responses when applying it to older persons with chronic illnesses. Specific considerations would be important during the selection of an appropriate data collection method (e.g., a structural face-to-face interview instead of a survey). Thirdly, the sample size of patients was small and has a limited representation of only the institutions involved in the study. The study is lacking generalizability across settings and countries; wider studies are recommended for the future, including older diabetes patients with experiences of inpatient settings as well. Finally, half of the data collection was conducted during the pandemic period when a rather large extent of routine health care services for chronically ill patients were suspended or provided by different means (e.g., phone calls). This fact should be taken into consideration when interpreting and comparing the results of this study with any other sources.

## 5. Conclusions

Health professionals have a more positive perception in regard to individualized care support and provision for older diabetes patients than the patients themselves. Individual personal life preferences of the patient are at least discussed during care provisions for the older diabetes patients from both health professionals’ and patients′ points of view. Patient characteristics, such as the type of glucose-lowering therapy, education, and body mass index, make a difference for older diabetes patients in their understanding and experience of individuality in care.

A change needs to be made to provide better individualized care for older diabetes patients, and the next steps would be to interview patients to see how the care can be improved and what will motivate patients to be more active in their care planning and implementation. Careful consideration of individual patients’ values, knowledge, habits, and behaviors would assist and assure that their personal life situation is taken into account during care processes.

## Figures and Tables

**Table 1:** Reliability statistics for the Lithuanian version of the Individualized Care Scale–Nurse Version (ICS–Nurse) and Patient Version (ICS–Patient).

Individualized Care Scale	Cronbach’s Alpha	
	ICS–Nurse	ICS–Patient
A-ICS	0.90	0.96
A-ICS–Clinical	0.84	0.94
A-ICS–Personal	0.84	0.85
A-ICS–Decision	0.80	0.92
B_ICS	0.92	0.96
B-ICS–Clinical	0.85	0.94
B-ICS–Personal	0.79	0.77
B-ICS–Decision	0.84	0.90
Total	0.95	0.91

**Table 2:** Characteristics of the patient sample (N = 145).

Variables	
Age (mean ± SD; median)	71.9 ± 6.2; 70.0
(Range)	65–92
Disease duration (mean ± SD)	15.7 ± 10.0
(Range)	1–46
	% (**N**)
Gender	
Female	59.3 (86)
Male	40.7 (59)
Age (in years)	
65–70	51.7 (75)
≥71	48.3 (70)
Education level	
Degree (university or college)	18.6 (27)
Less than degree	81.4 (118)
Place of residence	
Urban	71.7 (104)
Rural	28.3 (41)
Type of current glucose-lowering therapy	
Tablets	51.0 (74)
Insulin injections	37.9 (55)
Combined therapy (oral antidiabetics and injectable medications)	11.1 (16)
Type of diabetes mellitus	
Type 1	11.1 (16)
Type 2	88.9 (129)
Years of diabetes diagnosis	
1–10	37.9 (55)
11–20	37.9 (55)
≥21	24.2 (35)
Glucose profile (HbA1c) in %, (N = 143)	
Less than 7	44.1 (63)
7–9	43.4 (62)
>9	12.6 (18)
Body mass index, (N = 142)	
<30	47.9 (68)
≥30	52.1 (74)

**Table 3:** Health professionals’ and patients’ assessments of individualized care at the ICS dimension and sub-scale level.

Individualized Care Scale—ICS	Health Professionals	Patients	*p*
	Mean (SD)	Mean (SD)	
A-ICS (Total)	4.00 (0.54)	3.51 (0.92)	<0.001
A-ICS–Clinical	4.13 (0.55)	3.55 (1.00)	<0.001
A-ICS–Personal	3.82 (0.78)	3.40 (1.03)	0.008
A-ICS–Decision	3.99 (0.55)	3.54 (0.97)	0.003
B-ICS (Total)	4.08 (0.54)	3.58 (8.71)	<0.001
B-ICS–Clinical	4.17 (0.51)	3.60 (0.92)	<0.001
B-ICS–Personal	3.95 (0.60)	3.45 (0.95)	<0.001
B-ICS–Decision	4.06 (0.61)	3.65 (0.92)	0.003

**Table 4:** Health professionals’ and patients’ assessments of the support of patient individuality: item-level analysis.

Items of the Individualized Care Scale	Health Professionals (Mean (SD))	Patients (Mean (SD))	*p*
Clinical situation			
A01—Feelings about illness/health condition	4.21 (0.83)	3.54 (1.16)	<0.001
A02—Needs that require care and attention	4.27 (0.74)	3.66 (1.11)	<0.001
A0—Chance to take responsibility as far as possible	4.16 (0.65)	3.62 (1.17)	0.002
A04—Identify changes in how they have felt	4.21 (0.56)	3.59 (1.14)	<0.001
A05—Talk with patients about fears and anxieties	4.03 (0.79)	3.39 (1.23)	<0.001
A06—Find out how their health conditions affect them	4.07 (0.68)	3.55 (1.15)	0.003
A07—What the illness/health condition means to them	3.96 (0.89)	3.54 (1.16)	0.018
Personal life situation			
A08—What kinds of things they do in their everyday life	3.87 (0.91)	3.49 (1.16)	0.040
A09—Previous experience of hospitalization	3.63 (0.95)	3.39 (1.15)	0.252
A10—Everyday habits	3.86 (0.92)	3.41 (1.21)	0.011
A11—Family take part in their care	3.94 (0.88)	3.35 (1.14)	<0.001
Decisional control over care			
A12—Instructions to patients	4.31 (0.64)	3.67 (1.18)	<0.001
A13—What they want to know about illness/health condition	4.21 (0.72)	3.66 (1.20)	0.003
A14—Patients’ personal wishes with regards to their care	3.96 (0.75)	3.59 (1.07)	0.021
A15—Help patients take part in decisions	4.00 (0.68)	3.66 (1.06)	0.064
A16—Encourage patients to express their opinions	4.06 (0.83)	3.59 (1.10)	0.003

**Table 5:** Health professionals and patients’ assessments of the provision of individualized care: item-level analysis.

Items of the Individualized Care Scale	Health Professionals (Mean (SD))	Patients (Mean (SD))	*p*
Clinical situation			
B01—Feelings about illness/health condition	4.13 (0.65)	3.58 (1.02)	<0.001
B02—Needs that require care and attention	4.20 (0.62)	3.71 (1.03)	0.001
B03—Chance to take responsibility as far as possible	4.03 (0.65)	3.69 (1.07)	0.061
B04—Identify changes in how they have felt	4.27 (0.58)	3.64 (1.09)	<0.001
B05—Talk with patients about fears and anxieties	4.19 (0.64)	3.50 (1.14)	<0.001
B06—Find out how their health conditions affect them	4.26 (0.69)	3.52 (1.05)	<0.001
B07—What the illness/health condition means to them	4.19 (0.68)	3.68 (1.07)	0.001
Personal life situation			
B08—What kinds of things they do in their everyday life	3.96 (0.75)	3.57 (1.04)	0.014
B09—Previous experience of hospitalization	3.77 (0.72)	3.43 (1.12)	0.045
B10—Everyday habits	3.99 (0.73)	3.47 (1.10)	0.001
B11—Family take part in their care	4.10 (0.78)	3.40 (1.18)	<0.001
Decisional control over care			
B12—Instructions to patients	4.20 (0.75)	3.82 (1.05)	0.018
B13—What they want to know about illness/health condition	4.13 (0.74)	3.84 (1.03)	0.103
B14—Patients’ personal wishes with regards to their care	4.10 (0.68)	3.66 (1.03)	0.002
B15—Help patients take part in decisions	4.09 (0.67)	3.63 (1.06)	0.002
B16—Encourage patients to express their opinions	4.10 (0.70)	3.67 (1.04)	0.003

**Table 6:** Patients’ assessments of individualized care at the ICS dimension and sub-scale levels in relation to the current type of glucose-lowering therapy.

Individualized Care Scale—ICS	Type of Therapy	Mean Rank	*p* *	Type of Therapy	Mean Rank	*p* *
A-ICS (Total)	1 3	43.07 56.75	0.057	2 3	33.92 43.16	0.115
A-ICS–Clin	1 3	43.33 55.53	0.089	2 3	34.19 42.22	0.170
A-ICS–Pers	1 3	42.77 58.13	0.032	2 3	33.89 43.25	0.108
A-ICS–Dec	1 3	42.92 57.44	0.043	2 3	33.55 44.41	0.063
B-ICS (Total)	1 3	42.20 60.75	0.010	2 3	32.98 43.16	0.048
B-ICS–Clin	1 3	42.43 59.69	0.016	2 3	33.39 44.97	0.022
B-ICS–Pers	1 3	42.59 58.97	0.022	2 3	32.99 46.34	0.034
B-ICS–Dec	1 3	42.42 59.75	0.016	2 3	33.21 45.59	0.022

* Mann–Whitney U test; 1—tablets (*n* = 74), 2—insulin injections (*n* = 55), 3—combined (*n* = 16).
